# Non-photochemical Quenching: From Light Perception to Photoprotective Gene Expression

**DOI:** 10.3390/ijms23020687

**Published:** 2022-01-08

**Authors:** Dandan Lu, Yi Zhang, Aihong Zhang, Congming Lu

**Affiliations:** 1 State Key Laboratory of Crop Stress Adaptation and Improvement, School of Life Sciences, Henan University, Jinming Avenue, Kaifeng 475004, China; ludandan@henu.edu.cn; 2 State Key Laboratory of Crop Biology, College of Life Sciences, Shandong Agricultural University, Taian 271018, China; zhangyi@sdau.edu.cn (Y.Z.); ahzhang@sdau.edu.cn (A.Z.)

**Keywords:** energy-dependent quenching, light perception, photoprotection, photoprotective genes, non-photochemical quenching

## Abstract

Light is essential for photosynthesis but light levels that exceed an organism’s assimilation capacity can cause serious damage or even cell death. Plants and microalgae have developed photoprotective mechanisms collectively referred to as non-photochemical quenching to minimize such potential damage. One such mechanism is energy-dependent quenching (qE), which dissipates excess light energy as heat. Over the last 30 years, much has been learned about the molecular mechanism of qE in green algae and plants. However, the steps between light perception and qE represented a gap in our knowledge until the recent identification of light-signaling pathways that function in these processes in the green alga *Chlamydomonas reinhardtii.* In this review, we summarize the high light and UV-mediated signaling pathways for qE in *Chlamydomonas*. We discuss key questions remaining about the pathway from light perception to photoprotective gene expression in *Chlamydomonas*. We detail possible differences between green algae and plants in light-signaling mechanisms for qE and emphasize the importance of research on light-signaling mechanisms for qE in plants.

## 1. Introduction

Photosynthesis in plants and algae is a biological process that converts light energy into chemical energy, which is used in the Calvin–Benson–Bassham cycle to assimilate CO_2_ and produce organic compounds. Light is required for photosynthesis. However, the supply of light changes under natural conditions and photosynthetic organisms are often exposed to excess light. Excess light leads to over-excitation of the photosystems, resulting in photodamage to the photosynthetic apparatus and possibly cell death. Plants and algae have therefore evolved a photoprotective mechanism called non-photochemical quenching (NPQ) to dissipate excess light energy as heat [1,2].

NPQ involves the thermal dissipation of excess absorbed energy through the deexcitation of singlet excited chlorophyll in photosystem II (PSII) and was originally defined based on both the relaxation kinetics of PSII components and their sensitivity to chemical inhibitors [2,3]. Several different processes contribute to NPQ and are differentiated based on the molecular players involved as well as on fluorescence relaxation kinetics [4]. These types of NPQ include qE, qH, qI, qM, qT, and qZ.

Energy-dependent quenching (qE), a major contributor to NPQ, occurs in the light-harvesting complexes of PSII (LHCII). qE involves rapid induction and relaxation [5,6]. qE responds to most short-term light stress and is both activated and deactivated rapidly (from seconds to minutes). The process of qE is driven by the acidification of the thylakoid lumen that occurs under excess light conditions [7]. This acidification modifies the pigment composition of LHCII via the xanthophyll cycle and activates specific qE protein effectors, such as PSBS and/or light-harvesting complex stress-related proteins (LHCSRs), in both green algae and plants [8,9,10]. In the active state, these protein effectors increase the energy dissipation capacity of LHCII via an unknown mechanism [11,12].

The recently identified antenna-quenching component qH protects the photosynthetic apparatus under stress conditions in Arabidopsis [13,14]. qH, a sustained form of antenna quenching, is considered to be a distinct NPQ component that is independent of PSBS, pH gradient, zeaxanthin, the serine/threonine kinase STN7, inactivation of the PSII core protein D1, and other qI processes. This photoprotective mechanism requires the plastid lipocalin LCNP and is prevented by the suppressor of quenching1 (SOQ1) under non-stress conditions. LCNP and the relaxation of qh1 (ROQH1) were recently proposed to play dosage-dependent antagonistic roles in protecting the photosynthetic apparatus and in maintaining the light-harvesting efficiency in plants [15].

Photoinhibitory quenching (qI) was previously defined as all mechanisms that result in the light-induced decrease in the quantum yield of PSII due to D1 photoinactivation [4,16]. qI, also known as sustained zeaxanthin, is associated with zeaxanthin retention. qI includes all components with slow relaxation kinetics, such as photoinhibition due to PSII photoinactivation and uncharacterized modes of sustained thermal dissipation [17]. qI is among the most slowly forming and slowly relaxing components of NPQ; depending on both photoinhibition and long-term photoprotective quenching, qI takes several hours or longer to relax [14,18].

qM is a blue light-dependent quenching mechanism induced by chloroplast movement [19,20]. However, chloroplast movement was recently shown to have little influence on the effectiveness of photoprotection under high-light conditions in “shade”-grown Arabidopsis. Therefore, the existence of a chloroplast movement-dependent component of NPQ and the influence of chloroplast movements on photoinhibition should be thoroughly reevaluated [21].

qT occurs under low-light conditions and depends on state transitions involving the movement of phosphorylated antenna proteins away from PSII [22,23]. qZ is a zeaxanthin-dependent NPQ process that does not require an acidic lumen or PSBS. Instead, qZ involves the binding of zeaxanthin to monomeric antenna proteins [24,25]. qZ relaxes more slowly than qE and takes several minutes to tens of minutes to turn on and off [24,26,27].

Due to the importance of NPQ and the rapid progress in understanding the underlying mechanisms, various aspects of NPQ have been reviewed over the past several years, such as light stress and photoprotection [28,29], NPQ mechanisms [2,30,31], the evolution of NPQ mechanisms and photoprotective antenna proteins [9,32,33,34], and the photoreceptor-dependent regulation of photoprotection [35].

In this review, we summarize what is known about the high light and UV-mediated signaling pathways for qE in *Chlamydomonas*. We discuss several key questions that remain about the processes between light perception and photoprotective gene expression in *Chlamydomonas*. Finally, we delve into possible differences between green algae and plants in response to excess light as well as the importance of research on light signaling for efforts to improve agricultural productivity by regulating qE.

## 2. Non-Photochemical Quenching Signaling Pathways in the Green Alga *Chlamydomonas reinhardtii*

### 2.1. Induction of Photoprotective Genes

qE is controlled by the photoprotective proteins LHCSRs and/or PSBS, which function as specific qE effectors [9]. The genes encoding these two photoprotective proteins are referred to as photoprotective genes or *qE* genes [36]. *C. reinhardtii* contains three genes encoding LHCSRs, namely *LHCSR1*, *LHCSR3.1*, and *LHCSR3.2*, and two genes encoding PSBS, namely *PSBS1* and *PSBS2*. *LHCSR* genes are present in green algae and the model moss *Physcomitrium* (*Physcomitrella*) *patens* but not in vascular plants [8,37,38]. *PSBS* genes are found throughout green algae in *P. patens* and in vascular plants [5]. Thus, vascular plants and green algae appear to employ different proteins to regulate photosynthetic light harvesting under excess light conditions.

The expression of *LHCSR* genes is induced in response to environmental cues in the green alga *C. reinhardtii*. However, the expression patterns of *LHCSR3* and *LHCSR1* are different. *LHCSR3* expression is induced by high light [8,36,39,40,41], Ca^2+^ [42], low CO_2_ [43], and nutrient starvation [44], whereas *LHCSR1* expression is induced by UV-B [45] and nutrient starvation [44]. These results suggest that the expression of *LHCSR* genes is regulated by the environment where the green alga *C. reinhardtii* lives. The blue-light photoreceptor phototropin (PHOT) is essential for effective *LHCSR3* gene expression and protein accumulation under high-light conditions [46,47]. The UV-B photoreceptor UVR8 can induce UV-dependent *LHCSR1* and *PSBS* gene expression and protein accumulation [41,48]. The PSBS protein accumulates in *Chlamydomonas* only in response to UV-B [48] and very strong white light [49,50]. UV-B induces the accumulation of high levels of PSBS and LHCSR1, and much lower levels of LHCSR3, whereas high light induces the accumulation of high levels of LHCSR3 and lower levels of HCSR1 and PSBS [48,49,50]. The induction of photoprotective genes by UV-B is independent of photosynthetic electron transfer, whereas the induction of photoprotective genes under high light depends on this process [40,48]. The distinct expression patterns of *PSBS*, *LHCSR1*, and *LHCSR3* under UV-B and high light point to the evolutionary divergence of these signaling pathways, which is likely associated with the different characteristics of these proteins [48].

### 2.2. High-Light Signaling Pathway

LHCSR3, the major qE effector in *C. reinhardtii*, primarily accumulates under excess light conditions. To gain insight into how this process is regulated, the light-color dependency (action spectrum) of qE induction between 400 nm and 720 nm was investigated in *C. reinhardtii* in Minagawa’s group [46]. Blue light was more effective than red light at inducing the qE response and LHCSR3 accumulation, suggesting that blue-light photoreceptors are involved in the accumulation of LHCSR3 in high light. To investigate whether blue-light photoreceptors are involved in inducing LHCSR3 accumulation, Petroutsos et al. (2016) compared the kinetics of NPQ, the action spectra of qE, and the induction of LHCSR3 accumulation in mutants lacking blue-light photoreceptors, namely the animal-like cryptochrome (aCRY) [46] and PHOT. This analysis revealed that PHOT controls qE by inducing the accumulation of LHCSR3 in high light. This control requires the perception of blue light by the LOV domains of PHOT; the induction of LHCSR3 through the kinase domain of PHOT; and light dissipation in photosystem II via LHCSR3. The *phot* mutants display severely reduced fitness under excessive light conditions, indicating that the sensing, utilization, and dissipation of light is a concerted process that plays a vital role in the acclimation of microalgae to environments with variable light intensities and PHOT is a central player in the photoprotection of *C. reinhardtii* [46].

The roles of various molecules in the sensing (by photoreceptors) and utilization (by photosynthetic complexes) of environmental light have long been unclear. The findings about the roles of PHOT described above uncover the molecular link between the sensing and utilization of light, the two essential functions of photosynthetic organisms. How does PHOT control the expression of *LHCSR3.1* and *LHCSR3.2*? It appears that the LOV domains of PHOT provide blue-light sensitivity, while the kinase domain of PHOT performs signal transduction, possibly via the initiation of a cyclic nucleotide monophosphate (cAMP and/or cGMP)-signaling cascade. Downstream of PHOT, this signal is integrated with another regulatory signal from the chloroplast that carries information about the amount of absorbed light that is not used for CO_2_ fixation. The chloroplast signal relies on photosynthetic electron transfer [40,42], although the precise nature of this signal remains elusive. These integrated signals regulate the expression of *LHCSR3.1* and *LHCSR3.2* [46]. However, the signaling pathway between PHOT and *LHCSR3* is not clear.

Minagawa’s group further revealed the possible signaling components between PHOT and *LHCSR3* [47]. The *phot* mutant does not express LHCSR3 [46], its chlorophyll is bleached within 16 h, and the cells do not survive exposure to high-light conditions (1000 µmol photons m^−2^ s^−1^) [47], suggesting that the high-light sensitivity of this mutant is suppressed by inactivation of the negative regulators of *LHCSR3* and/or *LHCSR1* gene expression. The authors took advantage of this high-light sensitivity to obtain mutants in the signal transduction pathways between PHOT and the *LHCSR3* transcription site. They identified two *phot* suppressor loci involved in qE quenching: de-etiolated 1 (*det1*) and damaged DNA-binding 1 (*ddb1*). When grown under low-light conditions (40 µmol photons m^−2^ s^−1^), the growth rates of the suppressors (*det1-1 phot*, *det1-2 phot*, and *ddb1 phot*) and *phot* mutants were similar to that of the wild type (WT), and the photosynthetic performance of the suppressors was similar to that of the original *phot* mutant. By contrast, under high-light conditions, the *phot* mutant did not survive and the growth of WT cells was retarded; however, the suppressors grew even more rapidly than WT cells, suggesting that the *det1* and *ddb1* mutations suppress the *phot* mutation and confer high-light tolerance. Moreover, the suppressors recovered the qE quenching capacity of the mutants. Thus, the increased tolerance to high-light conditions in the suppressors appears to be associated with their photoprotection ability. Through a combination of genetic, biochemical, and molecular analyses, the authors concluded that DET1 and DDB1 (two downstream components of PHOT) are involved in qE quenching and in the induction of photoprotective genes including *LHCSR1/3* and *PSBS* under high-light conditions.

Next, Minagawa’s group investigated how DET1 and DDB1 regulate qE gene expression. Based on a yeast two-hybrid analysis and an inhibitor assay, the authors concluded that DET1 and DDB1 are part of a protein complex containing CULLIN 4 (CUL4). They proposed that DET1 and DDB1 act as central mediators by forming an E3 ubiquitin ligase complex together with CrCUL4 (CRL4^DET1^). This complex suppresses the expression of qE genes in the dark by directly ubiquitinating its targets and promotes the expression of these genes under high-light conditions by inhibiting the activity of the E3 ligase complex CUL4–DDB1^DET1^ via the PHOT signal.

Two questions are then raised: the possible targets of the E3 ligase complex CUL–DDB1^DET1^ and the possible signaling components that act downstream of this complex. Krishna Niyogi’s group recently identified SPA1 and CUL4, the components of a putative E3 ligase, as critical factors in a signaling pathway that controls the light-induced expression of qE genes. The authors proposed that the E3 ubiquitin ligase SPA1/COP1 acts upstream of CONSTANS (CrCO), a transcription factor that controls the qE capacity via cis-regulatory CrCO-binding sites in photoprotective genes [36]. Indeed, Jun Minagawa’s group showed that the transcription factors CrCO and Nuclear transcription Factor Ys (NF-Ys) form a complex that governs light-dependent photoprotective responses in *C. reinhardtii*. Moreover, they determined that the signal from the light perception to the CONSTANS/NF-Ys’ complex is directly inhibited by the E3 ubiquitin ligase SPA1/COP1 [41]. We note that in both studies, no accumulation of LHCRS1 or PSBS was induced by high light or UV in the *crco* mutants, while only a small amount of LHCRS3 accumulated in high light but not in UV [36,41], suggesting that CrCO is a key transcription factor controlling qE gene expression. Thus, CrCO likely functions downstream of the E3 ligase complex CUL4–DDB1^DET1^, although other transcription factors might be involved in regulating qE-associated genes as well.

**Figure 1:**
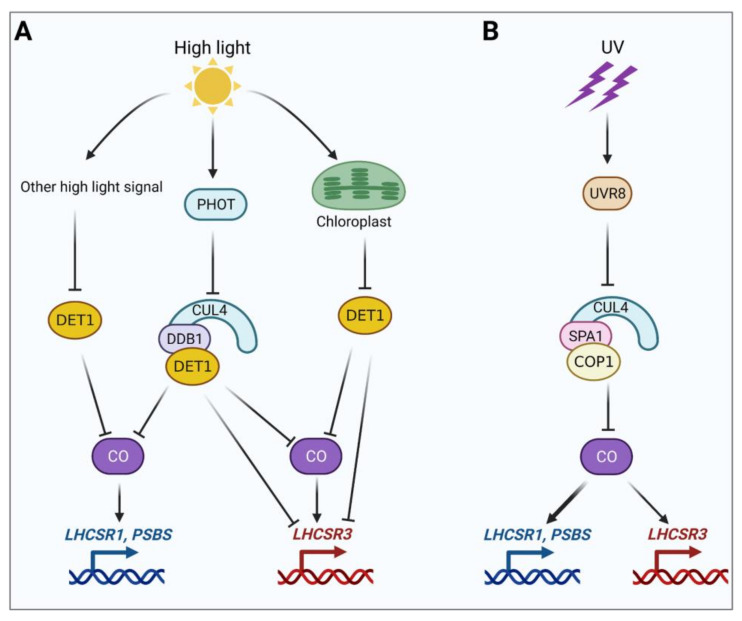
Models for the induction of *LHCSR1*, *LHCSR3*, and *PSBS* expression by high light and UV-B in *C. reinhardtii*. (**A**) Induction of *LHCSR1*, *LHCSR3*, and *PSBS* expression by high light via the putative E3 ligase complex CUL4-DDB1^DET1^ and other DET1-dependent high-light signaling pathways. (**B**) Induction of *LHCSR1*, *LHCSR3*, and *PSBS* expression by UV-B via the SPA1/COP1-dependent E3 ligase complex. See the main text for a discussion of the related signaling pathways. This figure was created with the help of the Biorender.

To reveal the possible high-light signaling pathways, Aihara et al. (2019) investigated the transcript patterns of *LHCSR1/3* and *PSB1/2* in the *phot* mutant as well as the suppressors under high-light conditions [47]. High light induced a significant increase in the transcript levels of *PSB1/2*, *LHCSR1*, and *LHCSR3.1/3.2* in the WT, whereas this high light-induced upregulation was suppressed in the *phot* mutant. These results reveal that there are at least two high light-induced signaling pathways for photoprotective genes: one that is PHOT-dependent and one that is PHOT-independent. Indeed, high levels of *PSB1/2* and *LHCSR1/3* transcripts were detected in both dark and high-light conditions in the *det1 phot* mutants. By contrast, in the *det1 phot* mutants, *PSB1/2* and *LHCSR1/3* were expressed at low levels in the dark but at high levels under high-light conditions, suggesting that the additional high-light signals are DET1-dependent. Therefore, *LHCSR1* and *PSBS1/2* expression is independent of active photosynthesis, whereas *LHCSR3* expression is dependent on active photosynthesis, suggesting that there are two additional DET1-dependent high-light signaling pathways: one that is independent of active photosynthesis (for *LHCSR1* and *PSBS1/2* expression) and one that is dependent on active photosynthesis (for *LHCSR3* expression; see Figure 1A).

It should be noted that the accumulation of LHSCR3 but not LHSCR1 or PSBS was induced by high light in the *crco* and *crco spa1-1* mutants, pointing to the existence of an SPA1/CrCO-independent but high light-dependent pathway [36]. Notably, the phenotypes of the *phot* and *crco* mutants are similar, suggesting that PHOT and CrCO might act in the same pathway. Thus, PHOT might act as a photoreceptor in the SPA1/CrCO-independent pathway. It is also possible that the SPA1/CrCO-independent pathway is under the same regulation as the chloroplast-DET1-dependent high-light signaling pathway (see Figure 1A).

The blue light-dependent PHOT-signaling pathway is not limited to green algae but appears to be common to other photosynthetic organisms. This pathway might have been acquired during their evolution in a blue light-dominated environment, such as water columns. NPQ in cyanobacteria is also triggered by strong blue light [51,52]. Several lines of evidence point to a link between blue light and photoprotection in diatoms, although the underlying mechanism remains elusive [53,54,55,56,57].

### 2.3. UVR8-Signaling Pathway

UV-B is the most energy-rich component of sunlight and is potentially damaging to organisms. For example, UV-B has significant effects on photosynthetic processes [58]; photodamage peaks in the UV-B part of the spectrum [59,60]. In *Chlamydomonas*, various nucleus-encoded photoprotective genes, such as *PSBS*, *LHSCR1*, and *LHSCR3*, are induced by exposure to low doses of UV-B [45]. These findings indicate that there is a direct link between signaling by the UV-B photoreceptor UVR8 and photoprotection in *Chlamydomonas*. Indeed, under UV-B conditions, UVR8 and its partner COP1 can initiate a signaling pathway that induces the expression of photoprotective genes which strongly contributes to photoprotection under high light. Moreover, the competence for qE induced by acclimation to UV-B strongly contributes to photoprotection upon subsequent exposure to high light, suggesting that the UV-B signal might act as a proxy for high light, priming the cells for photoprotection [48].

Which factors mediate the induction of *PSBS* and *LHCSR1* gene expression by the UVR8 signal? Several Deficient-in-*LHCSR*-expression (*DSR*) mutants (with reduced *LHCSR* gene expression) were obtained through a genetic screening via a bioluminescence reporter assay in Minagawa’s group [61]. Four mutants were recently characterized, including mutants of *DSR10* and *DSR15* with a mutation in *CONSTANS* and mutants of *DSR28* and *CC4286* with a mutation in *NF-YB* [41]. Compared to the WT (*LHCSR1*-*Luc717*), the *DSR15* (*crco-2*) and *DSR28* (*nfyb-1*) mutants exhibited pigment bleaching, a strong decrease in the maximal efficiency of PSII, an almost complete loss of qE capacity, and undetectable levels of key photoprotective proteins including LHCSR1, LHCSR3, and PSBS following exposure to excess light (including a low dose of UV-B), suggesting that both CrCO and NF-YB are essential for the functional activation of qE-dependent photoprotection in *C. reinhardtii*. The authors discovered that CrCO, NF-YB, and NF-YC interact with each other to form a CO/NF-YB/NF-YC complex and that all photoprotective genes (including *LHCSR1*, *LHCSR3.1/3.2*, and *PSBS1/2*) contain at least one CO-responsive element (CORE; CCACA22) as well as an NF-Y cis-element (CCAAT12) in the regions upstream of their start codons. Moreover, NF-YB associated with the promoters of the photoprotective genes in low light and this association appeared to be facilitated by exposure to high light. Considering the binding features of plant NF-YB/NF-YC in *A. thaliana*, these findings suggest that NF-YB weakly associates with the promoter regions of photoprotective genes under low light and that this association is reinforced by both CrCO accumulation and the formation of a CrCO/NF-YB/NF-YC complex under high light. Thus, the CrCO/NF-YB/NF-YC complex acts as a transcriptional module involved in regulating the expression of photoprotective genes in *C. reinhardtii*.

Then, Minagawa’s group further investigated the possible mechanism for the accumulation of CrCO, finding that the SPA1/COP1-dependent E3 ubiquitin ligase is involved in the degradation of this protein [41]. To clarify the link between the E3 ubiquitin ligase, CrCO accumulation, and photoprotective proteins, they treated various lines with the proteasome inhibitor MG132. Treatment of the *crco-2*/*CrCO* line with MG132 resulted in the accumulation of CrCO even under low-light conditions. The overaccumulation of photoprotective proteins (mainly LHCSR1 and PSBS) was also observed in the *spa1* mutant even under low-light conditions due to the accumulation of CrCO. These findings indicate that the SPA1/COP1 modules function in the photoprotection in *C. reinhardtii* by controlling the degradation of CrCO. Under low-light conditions, CrCO is degraded by the proteasome following its ubiquitination by the SPA1/COP1-dependent E3 ubiquitin ligase complex, thus inhibiting qE gene expression. However, upon exposure to UV-B, during which UVR8 deactivates the E3 ubiquitin ligase through the formation of the UVR8/SPA1/COP1 protein complex, the degradation of CrCO is inhibited and qE gene expression is activated [41] (see Figure 1B).

### 2.4. CrCO in the Regulation of Photoprotection

Dr. Minagawa’s and Dr. Niyogi’s groups simultaneously discovered that qE activity was almost completely absent in *crco* mutants, suggesting that CO is essential for the activation of qE-dependent photoprotection in *C. reinhardtii* [36,41]. The expression of *LHCSR1*, *PSBS*, and *LHCSR3* was significantly induced by both high light and UV in WT cells but was not detected in the *crco-2* mutant [41]. Similar results were obtained in another *crco* mutant, except that the expression of *LHCSR3* was not detected in low light but was detected in high light at a relatively low level comparable to that in the WT under low-light conditions [36]. Moreover, CrCO was shown to function as a transcription factor that regulates the expression of photoprotective genes [36,41]. These results indicate that the expression of photoprotective genes is tightly controlled by CrCO, although an SPA1/CrCO-independent but high light-dependent pathway might exist [36]. Thus, CrCO is thought to play a central role in the regulation of photoprotection. Moreover, the high light and UV-B-signaling pathways might regulate the expression of photoprotective genes by converging on CrCO, although an SPA1/CrCO-independent but high light-dependent pathway might also exist.

Based on recent achievements in elucidating high light and UV-B-signaling pathways, we propose models for high light and UV-B-induced *LHCSR1*, *LHCSR3*, and *PSBS* expression in *C. reinhardtii* (Figure 1).

### 2.5. Activation of Photoprotection by Different Wavelengths of Light

As discussed above, NPQ is activated through wavelength-specific light-signaling pathways mediated by PHOT (blue light) and UVR8 (UV-B) photoreceptors in the unicellular green alga *C. reinhardtii*. What is the biological significance of the activation of photoprotection by different wavelengths of light? It appears that LHCSR1, PSBS, and LHCSR1 have different action spectra: LHCSR1 and PSBS proteins mainly accumulate under UV light, whereas LHCSR3 accumulates under blue and red light. Moreover, UV-illumination at relatively low intensity activates NPQ more rapidly compared to blue or red light at relatively high intensity. This is because UV treatment induces photoprotective gene expression and protein accumulation at a significantly faster rate and with greater magnitude compared to blue or red-light treatment. UVR8 is responsible for the UV-dependent rapid activation of NPQ; this type of photoprotection is indispensable in *C. reinhardtii* under high-light conditions in the absence of LHCSR3, a photoprotective effector that is primarily activated via blue-light perception [62]. These findings suggest that the UVR8-dependent rapid activation of photoprotection might represent a complementary mechanism under conditions where blue light-induced photoprotection is not sufficient to dissipate excess light energy. In addition, the UVR8-dependent rapid activation of photoprotection may function as “preemptive photoacclimation” prior to the “subsequent photoacclimation” enabled by high light-dependent photoprotection [45,48,62].

## 3. Questions and Perspectives

Various aspects of photoprotection have been uncovered in recent decades through molecular, biochemical, biophysical, structural, genetic, and physiological studies. Here, we summarized the recent progress in elucidating the signaling pathways from light perception to photoprotective gene expression in the green alga *Chlamydomonas reinhardtii*. The progress highlighted here clearly demonstrates a link between light perception and photoprotection. We believe that the discovery of such a link will strengthen the connection between the research areas of photobiology and photosynthesis. The next step is to investigate how algae and plants sense color to regulate photoprotection and photosynthesis, a topic that has previously been ignored.

The discovery that several signaling pathways regulate the expression of photoprotective genes in *Chlamydomonas* (Figure 1) has prompted several questions. For example, what is the intimate link between the UVR8-dependent pathway and the high light-dependent pathway? Is there a switch that balances the UVR8-dependent pathway and PHOT-dependent pathway under natural sunlight conditions? Why (and how) are photoprotective genes induced at a significantly faster rate and with greater magnitude under UV treatment compared to blue or red light treatment? Why is the UVR8-dependent pathway independent of photosynthesis, whereas the PHOT-dependent pathway is dependent on this process? What is the nature of the photosynthetic signal and how is it integrated with the photosynthesis-dependent pathways to regulate LHCSR3 activity? Answering these questions will advance our knowledge of how light signaling regulates photoprotection in *Chlamydomonas*.

Although several light-signaling pathways for photoprotection have been deciphered in *Chlamydomonas*, how plants recognize and sense light color to regulate photoprotective gene expression is completely unknown. Moreover, our knowledge about how light signaling regulates photoprotection and the related genetic mechanisms in plants is still relatively limited. We anticipate exciting discoveries about how light signaling regulates qE in plants. It appears that green algae and plants may employ different light-signaling mechanisms in response to excess light. For example, *Chlamydomonas* contains five photoprotective genes (*LHCSR1*, *LHSCR3.1*/*3.2*, and *PSBS1*/*2*), whereas Arabidopsis has only two (*PSBS1*/*2*). In addition, in *Chlamydomonas*, as discussed above, CrCO is a key transcription factor that regulates the expression of photoprotective genes under high-light and UV conditions, and is central to the regulation of photoprotection, whereas CO and its ubiquitination mediate photoperiodic flowering and are crucial for the regulation of photoperiodic flowering in Arabidopsis [63,64]. Furthermore, *Chlamydomonas* has a distinct UV response compared to land plants: in *Chlamydomonas*, the UV response is initiated by relatively long wavelengths of UV light, including UV-A/B, whereas Arabidopsis preferentially senses relatively short wavelengths of UV (mainly UV-B/C) [62,65,66,67]. Therefore, it appears that the signaling pathway for photoprotection that depends on the blue-light photoreceptor PHOT in *Chlamydomonas* is not present in plants; this PHOT-dependent control of photoprotection was apparently lost during the colonization of land [46]. In Arabidopsis, PHOT mediates chloroplast relocation and qM-type NPQ under high-light conditions [19,68], although the existence of a chloroplast movement-dependent component of NPQ is in doubt [21]. However, it is possible that another response that is dependent on the blue-light photoreceptor CRY1 is involved in photoprotection, as CRY1 predominantly mediates the high light-induced expression of Arabidopsis *ELI1* and *2*, encoding EARLY LIGHT-INDUCED PROTEIN1 (ELIP1) and ELIP2 [69].

Photoprotection represents a promising target for crop improvement. Indeed, enhancing NPQ recovery by upregulating PSBS, violaxanthin de-epoxidase, and zeaxanthin epoxidase genes increased the quantum yield of CO_2_ assimilation, plant biomass, and yield in tobacco [70]. Enhancing the photoprotective capacity in rice by increasing PSBS protein levels resulted in enhanced biomass and grain yield [71]. Therefore, understanding the signaling pathway from light perception to photoprotective gene expression in plants including crops will be crucial for improving photoprotection by genetically exploiting photoprotection in plants exposed to sunlight and its intrinsic UV-B fraction. This, in turn, will enhance agricultural productivity. We look forward to seeing the light-signaling pathway that regulates NPQ in plants unveiled in the near future.

## Data Availability

Not applicable.
